# Does accountability for reasonableness work? A protocol for a mixed methods study using an audit tool to evaluate the decision-making of clinical commissioning groups in England

**DOI:** 10.1136/bmjopen-2015-007908

**Published:** 2015-07-10

**Authors:** Katharina Kieslich, Peter Littlejohns

**Affiliations:** Faculty of Life Sciences and Medicine, Division of Health and Social Care Research, King's College London, London, UK

**Keywords:** QUALITATIVE RESEARCH

## Abstract

**Introduction:**

Clinical commissioning groups (CCGs) in England are tasked with making difficult decisions on which healthcare services to provide against the background of limited budgets. The question is how to ensure that these decisions are fair and legitimate. Accounts of what constitutes fair and legitimate priority setting in healthcare include Daniels’ and Sabin's accountability for reasonableness (A4R) and Clark's and Weale's framework for the identification of social values. This study combines these accounts and asks whether the decisions of those CCGs that adhere to elements of such accounts are perceived as fairer and more legitimate by key stakeholders. The study addresses the empirical gap arising from a lack of research on whether frameworks such as A4R hold what they promise. It aims to understand the criteria that feature in CCG decision-making. Finally, it examines the usefulness of a decision-making audit tool (DMAT) in identifying the process and content criteria that CCGs apply when making decisions.

**Methods and analysis:**

The adherence of a sample of CCGs to criteria emerging from theories of fair priority setting will be examined using the DMAT developed by PL. The results will be triangulated with data from semistructured interviews with key stakeholders in the CCG sample to ascertain whether there is a correlation between those CCGs that performed well in the DMAT exercise and those whose decisions are perceived positively by interviewees. Descriptive statistical methods will be used to analyse the DMAT data. A combination of quantitative and qualitative content analysis methods will be used to analyse the interview transcripts.

**Ethics and dissemination:**

Full ethics approval was received by the King's College London Biomedical Sciences, Dentistry, Medicine and Natural and Mathematical Sciences Research Ethics Subcommittee. The results of the study will be disseminated through publications in peer review journals.

**Strengths and limitations of this study**

- Study designed to test the effectiveness of dominant frameworks for healthcare priority setting.
- Study designed to examine healthcare priority setting processes at a local (clinical commissioning group, CCG) level.
- Study designed to test the usefulness of a decision-making audit tool (DMAT) in evaluating decision-making processes.
- Study designed to identify current strengths and weaknesses of commissioning processes at a local level.
- Results will make an empirical contribution to the literatures on accountability for reasonableness (A4R), healthcare priority setting and organisational theory.

## Introduction

Policymakers worldwide are facing tight healthcare budgets. In the light of this, strategies for making inevitable priority setting decisions fair, legitimate and acceptable are sought at an academic and at a policy level. The accountability for reasonableness (A4R) framework by Daniels and Sabin is widely considered to represent one framework for fair and legitimate priority setting in healthcare.^1^ It is centred on the premise that it is easier to agree on fair *processe*s than on *principles* of fair decision-making in priority setting.^2^ Daniels and Sabin suggest that decision-making procedures that are transparent and consistent, and that provide public access to reasons for decision outcomes, will render priority setting decisions more acceptable and legitimate in the eyes of the individuals affected by them.^1^ They outline four conditions that priority setting has to meet in order to be considered fair and legitimate: (1) publicity, (2) relevance, (3) appeals and (4) enforcement. Publicity is about the transparency and accessibility of decisions. Under the ‘relevance condition’, reasons for decisions must be given. Under the ‘appeals condition’, processes that provide opportunities to challenge decisions must be in place. Finally, the ‘enforcement condition’ refers to mechanisms that ensure that the conditions 1–3 are upheld. Daniels and Sabin suggest that their claims are normative.^1^ They argue that if these conditions are implemented, then, over time, rationing and priority setting decisions in healthcare will become more legitimate and fair.

While A4R is a dominant framework in the literature on health prioritisation, it is not without its challenges or critics. On a practical level, Lauridsen and Lippert-Rasmussen criticise that A4R does not provide decision-makers with guidance as to how to distinguish between relevant and irrelevant grounds for making decisions.^3^ Owing to the difficult conceptual distinction between relevant and irrelevant reasons, a number of scholars now advocate an analysis of the content of decision-making to supplement the analysis of the processes of decision-making.^4^ The content of decision-making in health prioritisation is diverse, but it may include elements such as clinical effectiveness, cost-effectiveness and social values.^4^ Most notably, Clark and Weale have developed a framework that identifies the most important social values, that is, values that are held by a society at large, in relation to the content and the process of health prioritisation.^4^ This framework has been applied to priority setting processes in a number of countries including England,^5^ Germany^6^ and most recently Australia.^7^

On a conceptual level, Rid argues that A4R falls short of explaining the theoretical basis underpinning the conceptualisation of fairness.^8^ Additionally, despite Daniels’ and Sabin's view that A4R is compatible with theories of deliberate democracy^1^—in the sense that deliberation gives rise to relevant and irrelevant reasons for decisions—its relation to efforts of public participation remains opaque. That is to say that it is not entirely clear whether public participation is a necessity for meeting A4R conditions.

We would add to the list of criticisms that A4R neglects the conceptual and empirical insights provided by the literature on organisational and decision theory. Scholars in these fields have long examined the complexities of legitimacy and decision-making as multidimensional concepts. For example, Suchman illustrates that different types and subtypes of legitimacy exist and that organisations may choose different legitimation strategies depending on their goals and institutional contexts.^9^ Emphasising procedural fairness is not the only strategy that may be pursued by organisations. Moreover, Brockner *et al*^10^ outline a number of circumstances in which process fairness is not desirable for the agents or the recipients of decisions, which underlines our doubts that the focus on fair processes is sufficient in achieving legitimacy in healthcare priority setting.

Last but not least, the question whether A4R holds what it promises is under-researched. Does the fulfilment of the A4R conditions lead to more legitimate and fair decisions in the eyes of the individuals and stakeholders affected by them? During a series of roundtable discussions convened by the King's College London (KCL) and University College London (UCL) collaboration on social values in health priority setting, we spent a significant amount of time debating this question.^11^ Several members of the group pointed out that there is an intrinsic value in designing transparent and accountable priority setting processes, regardless of whether they are perceived as fair and legitimate by members of the public and stakeholders. However, given that Daniels and Sabin originally set out to address what they refer to as the legitimacy and fairness problems of managed care organisations (MCOs) in the USA vis-à-vis their insured members,^1^ the question of whether the fulfilment of A4R conditions influences the way affected individuals and groups view respective priority setting decisions deserves an empirical investigation.

So far, empirical research on A4R has focused on evaluations of whether healthcare organisations in different countries and at different levels of decision-making fulfil A4R conditions.^12–15^ It has been suggested that organisations such as the National Institute for Health and Care Excellence (NICE) in England and Wales and institutional processes of pharmaceutical benefit assessments more generally are examples of how A4R is implemented in practice.^13^ Indeed, Daniels’ and Sabin's original arguments around A4R centred on the adoption of new treatments, including pharmaceuticals, to be covered by MCOs in the USA.^1^ However, empirical studies of decision-making at a local level remain few. Moreover, while research has been carried out on whether A4R is a reasonable account of fair priority setting in the eyes of decision-makers, there is, to the best of our knowledge, no empirical account of whether the institutions that perform well according to the A4R conditions also perform well in the eyes of the individuals affected by their decisions. Against this background, we are conducting a study on the decision-making of clinical commissioning groups (CCGs) in England in which we use A4R as part of a wider conceptual framework to examine what factors contribute to fair and legitimate decisions at a local level.

Before we move on to provide details of our project, a brief account of our understanding of how to evaluate health priority setting decisions is in order as we seek to combine two of the most important schools of thought. While we accept the theoretical underpinnings of the A4R framework, we argue that its effectiveness in achieving legitimacy and fairness cannot be taken for granted. Like others,^12^ ^13^ ^15^ we are interested in the practical implications of A4R. Here we go beyond the normative assumptions of A4R in two ways. First, we expect that there are situations in which a decision-making authority might fulfil A4R conditions, and yet stakeholders may not buy into the way the decisions were made or they may continue to distrust the respective authority. For example, political affiliations or entrenched local alliances may prevent decisions from being perceived as fair and/or as legitimate. We argue that any account of a fair and legitimate decision-making process in health prioritisation must provide an opportunity to consider such scenarios, however conceptually and methodologically ‘messy’ they might be. The earlier mentioned literature on organisational research provides avenues for addressing this conceptual and methodological ‘messiness’, for example, by highlighting that different stakeholders may have different perceptions on what constitutes decision legitimacy in a given circumstance.

The second way in which we go beyond the assumptions entailed in A4R is related to our focus on content values. Along with an increasing number of our peers,^4^ we argue that the content is as important as the process of decision-making in health prioritisation. Accounts of the legitimacy of priority setting decisions remain incomplete without an account of the content values that play a role in arriving at decisions. As we shall see, we combine these views in a so-called decision-making audit tool (DMAT) that we use as a proxy for providing an account of both content and process values in priority setting.

Our study is a mixed methods study that examines the extent to which CCGs fulfil process and content criteria that are considered crucial in achieving legitimate and fair decisions in health. CCGs replaced Primary Care Trusts (PCTs) as the main institutions for commissioning healthcare at a local level in 2013. They make for good case studies for evaluating whether A4R and other accounts of legitimate healthcare decision-making actually result in decisions being perceived as more legitimate in the eyes of those affected by them. Our aim is to provide clinical commissioners and policymakers in England with a picture of the status quo of decision-making at a local level from which recommendations for further improvements might arise. The study will contribute to the emerging literature that looks at what it means to implement A4R.^3^ ^12^ ^14^ ^16^ It will also make an empirical contribution to the field of organisational research as it will rely on some of the conceptualisations proposed by organisational scholars to operationalise the concept of legitimacy in the analysis phase of the project.

## Research question and hypotheses

The research question arises from the previous overview of the dominant accounts of fair and legitimate priority setting in healthcare. The study addresses the following question:

Do CCGs that adhere to process and content values arising from frameworks of fair and legitimate priority setting produce more acceptable decisions in the eyes of key stakeholders?

There are three main hypotheses:

**H1**: Adherence to process and content values arising from theories of fair and legitimate priority setting leads to an increased legitimacy and acceptability of priority setting decisions in the eyes of key stakeholders.

**H2**: The decisions of those CCGs that adhere to process and content values will be perceived as more legitimate and acceptable by key stakeholders than those by CCGs that do not adhere to them or that do not make their decision-making criteria transparent.

**H3**: A DMAT to identify which values are addressed by a given healthcare organisation facilitates the organisation's and stakeholders’ awareness of its strengths and weaknesses and can provide strategies for improvement.

The operationalisation of the hypotheses will be detailed in the next section.

For the purpose of our research, ‘key stakeholders’ are those stakeholders who are directly involved in, or affected by, priority setting processes at the CCG level, for example, patient and service user groups, local advocacy groups, clinicians as well as members of the public.

## Methods/design

### The DMAT

On the basis of the A4R framework and on research emerging from an international collaboration on social values in health priority setting led by KCL and the UCL,^11^ we developed an educational intervention in the form of a DMAT which will be used as one mechanism to test the above hypotheses. The DMAT is based on Clark's and Weale's social values framework.^4^ The tool allows decision-makers and stakeholders to assess their organisational ‘decision-making profile’, which includes the identification of values and criteria that feature prominently in the decision-making process. Given that the literature on organisational research underlines the importance of alignment of an organisation’s activities with the values of the context in which it operates in order to achieve legitimacy,^9^ ^17^ the identification of values and criteria that inform current decision-making is a necessary step in addressing the question of whether these contribute to the legitimacy of decisions. In this way, the approach we propose is more comprehensive than A4R alone in that it incorporates process values *and* content values when evaluating an organisation's current approach to priority setting.

The audit tool consists of questions on a set of eight process and content values that have been identified in the extant literature on priority setting. An overview of the tool is presented in table 1. The process values include the institutional setting, transparency, accountability and participation while the content values include clinical effectiveness, cost-effectiveness, fairness and solidarity. Users of the audit tool are given a brief description of each of the values and a set of prompt questions to help them think about the implications of each. They are then asked to indicate their assessment of the extent to which they believe the audited healthcare institution adheres to the values in question. Responses are scored on a 1–5 Likert scale that reflects the respondents’ confidence in the adherence of the healthcare institution to the respective value. For example, on the value of transparency, respondents are asked the following audit question: On a scale of 1–5, how sure are you that your organisation can demonstrate that it offers understandable and accessible reasons for its decisions?

**Table 1:** The decision-making audit tool

	Description	Prompt questions	Audit question
*Process values*			
Institutional setting	Before you consider how best to respect social values and other criteria of decision-making, you need first to consider the role that your organisation (or the one you are auditing) plays in the wider institutional context of healthcare decision-making	What legal responsibilities does your organisation have with regard to healthcare resource allocation? What legal obligation is your organisation under to avoid discrimination, promote equality and diversity and match resources to population needs?	On a scale from 1 to 5, how sure are you that your organisation has systems in place to identify and address its legal responsibilities? (1 representing very unsure, 2 somewhat unsure, 3 undecided, 4 sure, 5 very sure)
Transparency	Those who commission healthcare are given considerable power and with power comes responsibility. Being transparent in their decision-making is one way in which organisations can assure themselves that they are not making decisions on grounds that are considered unfair or biased by the wider public	How clearly does your organisation offer reasons for decisions? When your organisation is faced with a difficult decision, has it been open about the difficulties with those who will ultimately be affected by the decisions?	On a scale from 1 to 5, how sure are you that your organisation can demonstrate that it offers understandable and accessible reasons for its decisions? (1 representing very unsure, 2 somewhat unsure, 3 undecided, 4 sure, 5 very sure)
Accountability	Those who commission healthcare have a great number of people and organisations to whom they are accountable. Sometimes accountability is formal, involving legal or financial accountability. Sometimes it is less formal, eg, to colleagues or local media outlets. In all cases, accountability requires an ability to give reasons for one's decisions	Has your organisation identified to whom it is formally and informally accountable? Does your organisation provide an account of the reasons for its decisions in a variety of formats so that those who are less used to reading long and complex documents can follow them?	On a scale from 1 to 5, how sure are you that your organisation can demonstrate that it is accountable? (1 representing very unsure, 2 somewhat unsure, 3 undecided, 4 sure, 5 very sure)
Participation	Participation of stakeholders and the wider public is important because it adds to the views and values that are considered when making decisions. Enabling different groups, eg, patients, the public, health professional and elected officials, to contribute to decision-making ensures that these different views are heard and special needs are understood	Whom does your organisation include in its decision-making process and how? What is the goal of the participation method your organisation has chosen (eg, deliberation, consultation, elicitation of public preferences)? How are the results of participation exercises incorporated in decision-making and how is this communicated to the participants?	On a scale from 1 to 5, how sure are you that your organisation can demonstrate that it ensures participation of relevant stakeholders and the wider public? (1 representing very unsure, 2 somewhat unsure, 3 undecided, 4 sure, 5 very sure)
*Content values*			
Effectiveness	Effectiveness is a necessary condition for the provision of good health and social care. No one should allocate resources to forms of care that do no good or do harm. However, knowing what is effective is not easy, especially in the absence of evidence in the form of clinical effectiveness studies in some areas of healthcare provision	Is there a system in place to identify the evidence for the effectiveness of commissioned services? How, and by whom, is effectiveness evidence being assessed and appraised?How are decisions made in the absence of evidence (note: absence of evidence is not the same as evidence of ineffectiveness)?	On a scale from 1 to 5, how sure are you that your organisation can demonstrate that it assesses effectiveness? (1 representing very unsure, 2 somewhat unsure, 3 undecided, 4 sure, 5 very sure)
Cost-effectiveness	Cost-effectiveness judgements centred on ‘value for money’ can be controversial. For some, it means that there is a risk that financial considerations could be put before patients’ needs. For others, it means that the needs of all patients, rather than a few, are considered and that the best possible care for the largest number of patients is secured	Is there a system in place to identify national guidance or standards such as NICE recommendations? Have you taken steps to assure that what you are commissioning is cost-effective? How are decisions made in the absence of evidence for cost-effectiveness?	On a scale from 1 to 5, how sure are you that your organisation can demonstrate that it assesses cost-effectiveness? (1 representing very unsure, 2 somewhat unsure, 3 undecided, 4 sure, 5 very sure)
Fairness	Fairness goes by different names. Some people talk about equity and others about human rights. In the area of healthcare prioritisation, fairness relates to the question whether all those who use healthcare services are treated with equal concern and respect	How are vulnerable patient groups identified in your area and how do you ensure adequate services for these groups? Are services commissioned only on the basis of need and not on other characteristics such as age, gender, ethnicity or sexual orientation?	On a scale from 1 to 5, how sure are you that your organisation can demonstrate that it is fair to all population and patient groups on whose behalf it is commissioning services? (1 representing very unsure, 2 somewhat unsure, 3 undecided, 4 sure, 5 very sure)
Solidarity	Solidarity is the principle that ‘we are all in it together’. This value implies that costs for healthcare will be covered collectively in order to secure access to healthcare for individuals	Are services accessible for all, eg, are there mechanisms in place to cover travel and other costs of access? Does your commissioning strategy create a situation in which some people have to fund elements of treatments from their own pockets in ways that are unduly burdensome?	On a scale from 1 to 5, how sure are you that your institution can demonstrate that it addresses the social value of solidarity? (1 representing very unsure, 2 somewhat unsure, 3 undecided, 4 sure, 5 very sure)

### Using the audit tool to assess CCG decision-making

We will employ the audit tool to assess the extent to which a sample of CCGs adheres to principles contained in the social values framework and, by extension, the A4R framework. The DMAT incorporates the social values framework in health priority setting and builds on the A4R framework. The audit tool can be used by healthcare decision-makers and key stakeholders alike, which means it can serve as an internal and an external audit tool. The tool will help elicit strengths and weaknesses of the current processes and content of CCG decision-making, which is frequently considered a key component in improving priority setting policies.

As a first step, we will develop ‘value profiles’ of CCG decision-making using the DMAT. In order to account for the fact that different kinds of decisions may give rise to different perceptions on what is necessary to achieve fairness and legitimacy, we will use the DMAT to assess both the mission statements, or equivalent, of our CCG sample—that is, how CCGs aspire to make decisions, as well as to assess specific types of decisions—that is, how criteria and values are used in practice. This approach reflects the complexity of CCG decision-making and ensures that a distinction is made between different types of decisions and the required legitimation processes. For example, a decision on whether to decommission a service is different from a decision on where to build a new hospital in that it may give rise to a different set of concerns by stakeholders, which in turn affects the way the legitimacy of the decision outcome is perceived. We will therefore test the usefulness of the DMAT in assessing a range of decisions that CCGs have made. When triangulating the results of this exercise with the results of the interviews, we may find that the fulfilment of A4R conditions is enough to achieve the status of legitimacy for some decisions, but not for others, for example, when decisions involve making moral judgements on funding healthcare such as those explored by Gkeredakis *et al*.^18^

The profiles of CCG decision-making will enable us to evaluate whether A4R and other criteria of fair and legitimate priority setting are met. This is a necessary step in operationalising hypotheses H1 and H2 because the current criteria for decision-making need to be identified in order to analyse whether they reflect A4R and other theories of fair priority setting. This will help us establish which CCGs perform well with regard to the principles contained in the previously outlined frameworks. The goal is to develop a comparative overview of the CCGs that have performed well in our audit exercise and those that have performed less well. We will then carry out semistructured interviews with stakeholders within the CCG sample, asking them about their general perceptions of the legitimacy of ‘their’ CCGs’ decisions and about their perceptions in specific examples of decision-making that they have been involved in. The interviews are the second step in operationalising H1 and H2 because they will provide us with insights into the views of those affected by the CCGs’ decisions on the legitimacy and fairness of CCG decision-making.

### Data collection

The application of the DMAT to publicly available CCG documents such as mission statements, terms of reference, minutes of meetings and other relevant documents will provide the first data set for analysis. A comparative analysis of the results of the decision-making audit will enable us to assess the relative position of CCGs in meeting principles of fair and legitimate priority setting. Informed by these results, we will conduct semistructured interviews with stakeholder groups within the catchment area of given CCGs.

The aim of the interviews is twofold. First, we aim to ascertain whether decision-makers and stakeholders believe that the current commissioning process in their area is (1) legitimate and acceptable, (2) if yes, why, and if no, why not and (3) what they believe would contribute to a greater legitimacy of local commissioning decisions. The interview protocol will include examples of recent decision-making in each CCG and follow-up questions will be asked during the interview. Second, we aim to gain an understanding of what the stakeholders believe characterises ‘legitimate’ and ‘fair’ priority setting processes in order to identify any potential additional conditions that need to be fulfilled in order for local decisions to be considered legitimate and fair. This will also allow us to identify themes that are connected to the political nature of local decision-making processes that we alluded to in the introduction. Moreover, it will allow us to ascertain whether the conceptualisations of legitimacy by stakeholders reflect one or more of the types of legitimacy that the organisational literature discusses.^9^

The results of the interviews will be compared with the results of the auditing process to elicit whether there is a correlation between the CCGs that performed well in the audit process and those whose legitimacy was perceived positively by the interviewees. In other words, by using the DMAT to outline the decision-making profile of CCGs and triangulating this with what the stakeholders think of their CCG, we hope to gain an understanding of whether the adherence to principles emerging from theories of legitimate and fair priority setting contributes to more legitimate decisions in the eyes of affected stakeholders. The comparative analytical process is thus the key step in operationalising H1 and H2.

In addition to the interviews with the stakeholders, we will facilitate cross-borough as well as local workshops in which we introduce the audit tool, assess its usability as an internal and external audit tool and further elicit stakeholder perceptions on what constitutes legitimate priority setting in healthcare. This is the main mechanism by which we will test hypothesis H3, namely that the DMAT is useful in facilitating an evaluation of current decision-making and outlining improvements for the future. In doing so, we hope to contribute to educational efforts to raise public awareness about the need for priority setting. By engaging the workshop participants in an audit exercise on their local CCGs, we will also gather data that will help us check whether the results of our own institutional audit are valid. Moreover, both the workshop and the interview stages will lead to a better understanding of what the indicators of legitimacy are according to stakeholders. These indicators can be used to empirically test and, if necessary, refine the conceptualisations of legitimacy that the literatures on A4R and organisational theory currently provide.

### Sampling methods

A pilot study of the project will be conducted on CCGs in south London. Following the pilot study, a random sampling technique will be used to sample a selection of CCGs across England on which the DMAT will be used.

Interviewees will be sampled using a combination of purposive and snowball sampling methods.

Workshops will be carried out primarily in south London. Owing to the fact that the purpose of the workshops is to test the usefulness of the DMAT (H3), the workshop participants do not necessarily have to be aligned with our sample of CCGs. CCGs, a wide array of patient and professional advocacy groups, local and national voluntary health organisations will be invited to participate in the workshops.

### Data analysis

The study employs a comparative case study approach. The results of the DMAT will be analysed and presented using methods of descriptive statistics. The interview transcripts will be coded according to principles arising from theories on legitimate and fair priority setting as well as from the conceptualisations found in the literature on organisational legitimacy. Any new themes will be coded as such. The data will then be analysed using a mix of qualitative and quantitative content analysis methods.

## Dissemination

The results of the study will be disseminated through publications in peer review journals. The results will also be shared with workshop and interview participants as well as the wider audience of CCGs, Health and Wellbeing Boards and advocacy groups such as Healthwatch.

## Policy implications

In addition to addressing an empirical gap in the current literature, this research is of relevance for policymakers, healthcare decision-makers and the public alike. The DMAT provides a means for stakeholders to take stock of their current work in priority setting, on the basis of which future priority setting strategies may be developed. The audit tool also provides a means for public representatives to audit their local or national healthcare organisations. If our research can demonstrate that the audit tool is useful, the tool might serve as a means to increase the effectiveness of public participation in priority setting processes. The tool has the potential of offering a practical way for the public to hold the decision-making authorities to account while simultaneously providing a means for these authorities to critically evaluate their own processes.

Moreover, our study on whether criteria of fair and legitimate priority setting are met by CCGs will identify current barriers to or facilitators of legitimacy at a local level. The identification of barriers and facilitators may contribute to outlining strategies for achieving fairer and more legitimate priority setting processes. Decision-makers are more likely to implement a framework that has been shown to be successful and effective in practice, and our study aims to examine if and how adherence to theories of fair priority setting leads to more acceptable decisions in the eyes of affected groups and individuals. We hope that this will provide insights into the effectiveness of frameworks such as A4R and the social values framework. If the results of our study indicate the effectiveness of these frameworks, we hope that this may encourage decision-makers such as commissioners to consider A4R conditions and social values more systematically than is currently visible at a local level.
